# Association between coronary heart disease and erectile dysfunction in Chinese Han population

**DOI:** 10.18632/oncotarget.15654

**Published:** 2017-02-23

**Authors:** Guo-Xiang Tian, Sheng Li, Tong-Zu Liu, Xian-Tao Zeng, Wan-Lin Wei, Xing-Huan Wang

**Affiliations:** ^1^ Department of Urology, Zhongnan Hospital of Wuhan University, Wuhan, China; ^2^ Center for Evidence-Based and Translational Medicine, Zhongnan Hospital of Wuhan University, Wuhan, China; ^3^ Department of Cardiology and 4th Cadres Ward, General Hospital of Beijing Military Command, Beijing, China

**Keywords:** case-control study, China, coronary heart disease, erectile dysfunction

## Abstract

To investigate the association between coronary heart disease (CHD) and erectile dysfunction (ED) in Chinese Han population. Patients who went to the andrological out-patient clinic of our hospital between August 1, 2015 and May 1, 2016 and met all eligible criteria were enrolled in this study. The patients diagnosed as ED using self-administered International Index of Erectile Function-5 (IIEF-5) questionnaire were considered as case group and others were considered as control. The cases were categorized as mild, moderate, and severe ED. Subjects were interviewed for the history of CHD. Uni- and multivariate logistic regression models were used to calculate odds ratios (ORs) and corresponding 95% confidence intervals (CIs) using the SPSS 18.0 software. A total of 240 participants (56 ED patients and 184 controls) were enrolled. CHD prevalence was higher in cases without statistical significance (OR = 1.20, 95%CI = 0.63-2.29; *p* = 0.58). Results of adjusted analysis also showed a non-significantly increased risk (OR = 1.25, 95%CI = 0.55-2.85; *p* = 0.59). Stratified analysis by severity of ED revealed similar results. This study suggests no significant association exists between CHD and ED in Chinese Han population.

## INTRODUCTION

Erectile dysfunction (ED) is a multifactorial disease and its prevalence increased with age. Currently, it is estimated that approximately 8 million men has been affected by ED in the USA [1], and the Massachusetts Male Aging Study reported that the incidence rate of ED in male from 40 years to 70 is about 52% [2]. Cardiovascular disease (CVD) is the leading cause of mortality in the worldwide [3]. There are many co-risk factors for ED and CVD, such as age, diabetes, tobacco smoking, alcohol consumption, periodontal disease, and obesity [4–6]. The possible association between ED and CVD has been investigated in numerous epidemiological studies. According to systematic reviews and meta-analyses based on these epidemiological studies, ED is associated with increased risk of CVD, among which the coronary heart disease (CHD) is of particular importance [7–10].

On the other hand, endothelial damages typically found in the cardiovascular system may also occur in the penis arteries, therefore ED might be considered as a complication of CVD or a long-term secondary consequence of systemic arterial disorders (especially CHD). In view of this specific consideration, it is tempting to speculate that CHD patients might increase the risk of ED compared with healthy population. The objective of the present study is to investigate the relationship between CHD and ED in Chinese Han population. This study was reported in accordance with the recommended STROBE (Strengthening the Reporting of Observational studies in Epidemiology) statement [11].

## MATERIALS AND METHODS

### Ethics statement

The following methods were carried out in accordance with the approved guidelines. Written informed consent was obtained from each patient, and the experimental protocols were approved by the Ethics Committee of the Zhongnan Hospital of Wuhan University.

### Study population

This study used a hospital-based case-control design. Patients who went to the andrological out-patient clinic of Zhongnan Hospital of Wuhan University between August 1, 2015 and May 1, 2016 were potential subjects of this study. The eligibility criteria were as follows: Han Chinese population, no mental disorders, capable of participating in interviews, willing to cooperate and to provide the informed consent. All authors reviewed the results and approved the final version of the manuscript. The cases were those diagnosed as ED during the study period according to the self-administered International Index of Erectile Function-5 (IIEF-5) questionnaire [12]. A translated Chinese language version [13] of this IIEF-5 questionnaire was used with a score of ≤21 being considered indicative of a ED diagnosis. We categorized ED into three levels by severity according to the IIEF scores: mild (score: 12 to 21), moderate (8 to 11), and severe (7 or less). A blinded questionnaire analysis was performed independently by two authors. Those clinic patients diagnosed free of ED were considered as controls. Meanwhile, subjects were interviewed for the history of CHD. The diagnosis of CHD was in accordance with clinical guidelines.

### Physical and laboratory examination

All subjects received a thorough physical and laboratory examination, including serum lipid profiling. Height and weight were measured for each subject. We then calculated the body mass index (BMI), and combined with the results of serum lipid profiling to judge whether hyperlipidemia was present. A detailed history of all cases and controls, including age, CHD, hypertension, diabetes, tobacco smoking, alcohol consumption, and physical activity status were recorded during study interviews.

### Statistical analysis

Means and standard deviations (SDs) were used to summarize age and IIEF-5 scores, whereas percentages were used to summarize data on tobacco smoking, alcohol consumption, hypertension, diabetes, CHD, hyperlipidemia, BMI, and physical activity. Pearson chi-square test or *t*-test was used to investigate the association between CHD and ED, and crude ORs with their 95% confidence intervals (CIs) were calculated. Then a multiple logistic regression analysis was performed to obtain adjusted ORs and their 95%CIs by adjusting for the following covariates: age, hypertension, BMI, smoking history, drinking history, physical activity, hyperlipidemia, and diabetes. All analyses were carried out using the SPSS 18.0 software and a two-side *p* ≤ 0.05 was considered statistically significant.

## RESULTS

**Figure 1:**
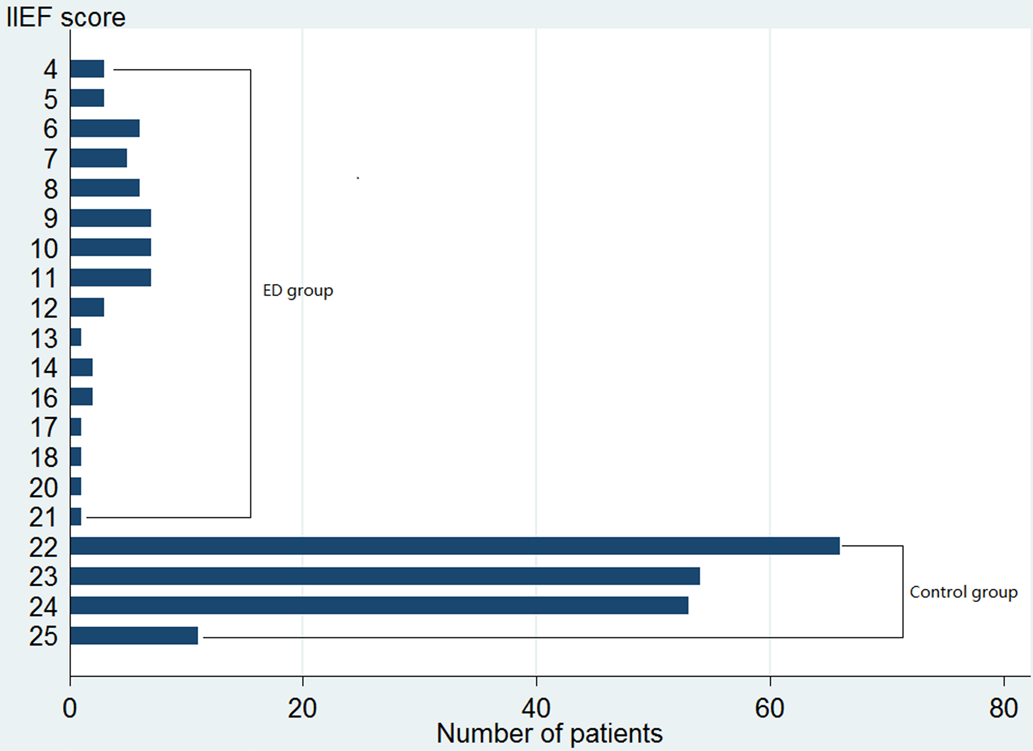
Distribution of results of scores of IIEF-5 of all cases and controls

A total of 240 participants with 56 ED patients and 184 controls were finally included. The age of diagnosis, score of IIEF-5 and percentage of hypertension cases were significantly higher among cases compared with controls, and the distribution of other variables was similar in the case and control group. Table 1 presents a summarization of socio-demographic characteristics, medical co-morbidities, and prevalence of CHD for cases and controls. Figure 1 shows the results of IIEF-5 scores for all cases and controls.

**Table 1:** Demographic characteristics of the study population

Parameters	Total (*n* = 240)	Patients with ED				Paitents without ED (*n*=184)	*P* value
		Total (*n*=56)	Mild ED (*n*=12)	Moderate ED (*n*=27)	Severe ED (*n*=17)		
Age (y)	49.8±13.1	55.8±12.9	53.8±12.2	54.2±12.6	59.8±13.6	48±12.7	<0.001
IIEF	19.9±6.0	9.7±3.8	15.4±3.1	9.6±1.1	5.8±1.1	23.0±0.9	<0.001
Smoking (%)	41 (17.1)	12 (21.4)	0 (0)	5 (18.5)	7 (41.2)	29 (15.8)	0.324
Driking (%)	31 (12.9)	8 (14.3)	1 (8.3)	4 (14.8)	3 (17.6)	23 (12.5)	0.727
Hypertension (%)	61 (25.4)	40 (71.4)	7 (58.3)	19 (70.4)	14 (82.4)	21 (11.4)	<0.001
Diabetes (%)	23 (9.6)	7 (12.5)	2 (16.7)	1 (3.7)	4 (23.5)	16 (8.7)	0.397
CHD (%)	70 (29.2)	18 (32.1)	6 (50.0)	5 (18.5)	7 (41.2)	52 (28.3)	0.576
Hyperlipidemia (%)	45 (18.8)	13 (23.2)	1 (8.3)	5 (18.5)	7 (41.2)	32 (17.4)	0.328
Body mass (%)							0.289
Normal BMI	140 (58.3)	31 (55.4)	8 (66.7)	15 (55.6)	8 (47.1)	109 (59.2)	
Overweight	85 (35.4)	19 (33.9)	4 (33.3)	12 (44.4)	3 (17.6)	66 (35.9)	
Obsity	15 (6.3)	6 (10.7)	0 (0)	0 (0)	6 (35.3)	9 (4.9)	
Physical activity (%)							0.977
None	129 (53.8)	30 (53.6)	5 (41.7)	14 (82.4)	11 (64.7)	99 (53.8)	
Occasionally	49 (20.4)	11 (19.6)	2 (16.7)	5 (18.5)	4 (23.5)	38 (20.7)	
Regularly	62 (25.8)	15 (26.8)	5 (41.7)	8 (29.6)	2 (11.8)	47 (25.5)	

Abbreviations: ED, erectile dysfunction; IIEF, International Index of Erectile Function.

CHD was found among 18 (32.1%) cases and 52 (28.3%) controls, the chi-square test indicated that cases had a non-significantly higher prevalence of CHD than controls (*p* = 0.58), and the OR was 1.20 (95%CI = 0.63-2.29). The crude OR was 2.54 for mild ED (95%CI = 0.78-8.23; *p* = 0.12), 0.58 for moderate ED (95%CI = 0.21-1.60; *p* = 0.29), and 1.78 for severe ED (95%CI = 0.64-4.92; *p* = 0.27), respectively. After the adjustment for age, hypertension, BMI, smoking history, drinking history, physical activity, hyperlipidemia, and diabetes, the results showed that CHD might increase the risk of ED for 1.25 times (OR = 1.25, 95%CI = 0.55-2.85; *p* = 0.59) in overall ED patients, but the effect was probably non-significant. Stratified analysis by severity of ED revealed similar results (Table 2).

**Table 2:** Results of univariate and multivariate logistic regression analyses for risk factor of CHD in association with ED

Severity of ED	No. of ED	No. of CHD	OR (95%CI)	*P* for OR	ad-OR* (95%CI)	*P* for ad-OR
Without ED (controls)	184	52	Reference [1.0]	-	Reference [1.0]	-
Total ED	56	18	1.20 (0.63-2.29)	0.58	1.25 (0.55-2.85)	0.59
Mild ED	12	6	2.54 (0.78-8.23)	0.12	4.08 (0.97-17.2)	0.06
Moderate ED	27	5	0.58 (0.21-1.60)	0.29	0.42 (0.12-1.38)	0.15
Severe ED	17	7	1.78 (0.64-4.92)	0.27	4.07 (0.88-18.78)	0.07

*covariates were: age, hypertension, body mass index, smoking history, drinking history, physical activity, diabetes.

## DISCUSSION

ED is defined as consistent inability to reach and maintain an erection satisfactory for sexual activity [14]. The penile circulation involves smaller arteries and this may explain the early clinical manifestation of ED [10], hence, this can also prompt that the function of penis might be influenced by CHD. In 2000, “The Massachusetts Male Aging Study” for the first time revealed that CHD and CHD risk factors (including hypertension, diabetes, dyslipidemia and smoking) were associated with the prevalence of ED [15]. Our study based on 240 Chinese Han subjects investigated the association between CHD and ED, and the results indicated that CHD increased the risk of ED but the effect was non-significant. Results adjusted for co-risk factors of ED and CHD i.e. the hypertension, diabetes, dyslipidemia, alcohol, BMI, physical activity, and smoking, showed that compared with unadjusted results, the ORs were increased; however, all the 95%CIs include 1.00 (Table 2).

Normal penile erection depends on the coordination of psychological, endocrine, vascular, and neurological systems [16]. ED is considered as a part of poly vascular disease [17], and endothelial dysfunction is the common denominator to many vascular risk factors that can lead to ED [18]. Epidemiological studies indicated that hypertension, atherosclerosis, cigarette smoking, diabetes, hyperlipidemia, and pelvic irradiation were associated with penilearterial insufficiency [19]. As we know, hypertension and atherosclerosis are associated with the onset and development of CHD. Studies have confirmed a significantly higher incidence and prevalence of ED in patients with hypertension [20]. Moreover, ED and CHD share common risk factors, with endothelial dysfunction being an important underlying pathological change in both ED and CHD [21]. Hence, we can speculate that CHD patients might have high risk of developing ED. Atherosclerotic plaque is often found in the CHD patients. Since penile arteries were quite small, any atherosclerotic plaques located within these arteries may easily block them and then result in ED.

As we know, the ED has been found to frequently precede the onset of CHD and represents an early marker of CHD, which has been written in guidelines [22]. Our results showed no difference in the presence of CHD cases in the ED and non-ED group. This might disprove ED as a strong predictor of CHD. However, CHD patients might have higher risk of ED onset and development. Our study with a relatively small sample of 240 participants might not have the power to identify the real relationship between CHD and ED. In 2015, Montorsi and colleagues reported a typical case of a patient with ED developing after acute coronary event [23]. Besides, there are five organic causes of ED: neurogenic ED, endocrinological ED, vasculogenic ED, drug-induced ED, and ED due to aging, lifestyle factors, and systemic diseases [20]. Hence, the CHD might primarily influence the vasculogenic ED. However, we cannot distinguish the type of ED in our current study. The real relationship of CHD and vasculogenic ED remains to be investigated in the future.

The major strength of our study is that we have stratified the ED into mild, moderate, and severe. The results showed an increased risk for mild and severe ED and a decreased risk for moderate ED. This phenomenon puzzled us and we thought this might be caused by small sample size, or fewer vasculogenic ED subjects in the moderate group. The mechanism needs to be further explored and discussed. In future studies, we recommend to record and report the relationship between CHD and different type of ED, besides the severity of ED. Moreover, our study focused on the Chinese Han population in the middle China mainland. For the socio-economic conditions also a risk factor for both ED and CHD [24–25], the results of our study is not adequately representative for the entire Chinese Han population. Since diverse socio-economic conditions and numerous ethnicities exist over mainland China, relevant studies for other areas and ethnicities are in need.

In summary, this study indicates that the CHD prevalence is similar in the ED group and control group in Chinese Han population. However, due to the limitations of this study, we suggest performing large sample size studies for further identification. Besides, the possible mechanism of the interaction between CHD and ED remains to be explored.

## References

[1] Selvin E, Burnett AL, Platz EA (2007). Prevalence and risk factors for erectile dysfunction in the US. Am J Med.

[2] Feldman HA, Goldstein I, Hatzichristou DG, Krane RJ, McKinlay JB (1994). Impotence and its medical and psychosocial correlates: results of the Massachusetts Male Aging Study. J Urol.

[3] Lloyd-Jones D, Adams RJ, Brown TM, Carnethon M, Dai S, De Simone G, Ferguson TB, Ford E, Furie K, Gillespie C, Go A, Greenlund K, Haase N (2010). Executive summary: heart disease and stroke statistics—2010 update: a report from the American Heart Association. Circulation.

[4] Leng WD, Zeng XT, Kwong JS, Hua XP (2015). Periodontal disease and risk of coronary heart disease: An updated meta-analysis of prospective cohort studies. Int J Cardiol.

[5] Montorsi P, Ravagnani PM, Galli S, Rotatori F, Briganti A, Salonia A, Deho F, Montorsi F (2004). Common grounds for erectile dysfunction and coronary artery disease. Curr Opin Urol.

[6] Keller JJ, Chung SD, Lin HC (2012). A nationwide population-based study on the association between chronic periodontitis and erectile dysfunction. J Clin Periodontol.

[7] Yamada T, Hara K, Umematsu H, Suzuki R, Kadowaki T (2012). Erectile dysfunction and cardiovascular events in diabetic men: a meta-analysis of observational studies. PLoS One.

[8] Dong JY, Zhang YH, Qin LQ (2011). Erectile dysfunction and risk of cardiovascular disease: meta-analysis of prospective cohort studies. J Am Coll Cardiol.

[9] Guo W, Liao C, Zou Y, Li F, Li T, Zhou Q, Cao Y, Mao X (2010). Erectile dysfunction and risk of clinical cardiovascular events: a meta-analysis of seven cohort studies. J Sex Med.

[10] Gandaglia G, Briganti A, Jackson G, Kloner RA, Montorsi F, Montorsi P, Vlachopoulos C (2014). A systematic review of the association between erectile dysfunction and cardiovascular disease. Eur Urol.

[11] Vandenbroucke JP, von Elm E, Altman DG, Gotzsche PC, Mulrow CD, Pocock SJ, Poole C, Schlesselman JJ, Egger M, Initiative S (2014). Strengthening the Reporting of Observational Studies in Epidemiology (STROBE): explanation and elaboration. Int J Surg.

[12] Rosen RC, Cappelleri JC, Smith MD, Lipsky J, Pena BM (1999). Development and evaluation of an abridged, 5-item version of the International Index of Erectile Function (IIEF-5) as a diagnostic tool for erectile dysfunction. Int J Impot Res.

[13] Chung SD, Chen YK, Liu SP, Lin HC (2013). Association between ED in ankylosing spondylitis: a population-based study. Int J Impot Res.

[14] Lue TF (2000). Erectile dysfunction. N Engl J Med.

[15] Feldman HA, Johannes CB, Derby CA, Kleinman KP, Mohr BA, Araujo AB, McKinlay JB (2000). Erectile dysfunction and coronary risk factors: prospective results from the Massachusetts male aging study. Prev Med.

[16] Prieto D (2008). Physiological regulation of penile arteries and veins. Int J Impot Res.

[17] Katsiki N, Wierzbicki AS, Mikhailidis DP (2015). Erectile dysfunction and coronary heart disease. Curr Opin Cardiol.

[18] Virag R, Bouilly P, Frydman D (1985). Is impotence an arterial disorder? A study of arterial risk factors in 440 impotent men. Lancet.

[19] Jackson G (2007). The importance of risk factor reduction in erectile dysfunction. Curr Urol Rep.

[20] Shamloul R, Ghanem H (2013). Erectile dysfunction. Lancet.

[21] Inman BA, Sauver JL, Jacobson DJ, McGree ME, Nehra A, Lieber MM, Roger VL, Jacobsen SJ (2009). A population-based, longitudinal study of erectile dysfunction and future coronary artery disease. Mayo Clin Proc.

[22] Nehra A, Jackson G, Miner M, Billups KL, Burnett AL, Buvat J, Carson CC, Cunningham GR, Ganz P, Goldstein I, Guay AT, Hackett G, Kloner RA (2012). The Princeton III Consensus recommendations for the management of erectile dysfunction and cardiovascular disease. Mayo Clin Proc.

[23] Montorsi P, Ravagnani PM, Vlachopoulos C (2015). Clinical significance of erectile dysfunction developing after acute coronary event: exception to the rule or confirmation of the artery size hypothesis?. Asian J Androl.

[24] Allen K, Pearson-Stuttard J, Hooton W, Diggle P, Capewell S, O’Flaherty M (2015). Potential of trans fats policies to reduce socioeconomic inequalities in mortality from coronary heart disease in England: cost effectiveness modelling study. BMJ.

[25] Martin AL, Huelin R, Wilson D, Foster TS, Mould JF (2013). A systematic review assessing the economic impact of sildenafil citrate (Viagra) in the treatment of erectile dysfunction. J Sex Med.

